# How and Why Does a Fly Turn Its Immune System Off?

**DOI:** 10.1371/journal.pbio.0050247

**Published:** 2007-09-18

**Authors:** David S Schneider

## Abstract

The fly immune response is actively turned down, and if it is not, pathology results.

With its powerful genetics and rapid generation time, the fruit fly, Drosophila, provides crucial insights into the evolution and conservation of fundamental immune mechanisms. The best example of molecules discovered in the fly that are shared in human immunity operate in the Toll signaling pathway, one of first lines of defense against pathogens. Drosophila can also benefit human health by providing a model for immune systems of arthropod vectors of infectious disease. Additionally, from a comparative immunological perspective, it is useful to know how different organisms have evolved solutions for fighting infections, because this information can lead to new ways of thinking about disease.

At a simple level, it is clear that many immune responses are designed to turn on when needed, presumably because their constitutive expression would be deleterious. After induction, immune responses must also be turned off, for the same reason they are not left on from birth. The resolution of inflammation in humans occurs through a complex active signaling program [1]. This negative regulation is important because excessive inflammation leads to pathology and death. The fly served as a good model for the induction of an innate immune response, pointing to conserved pathways and mechanisms. Recent work shows that the fly's immune response is more subtly regulated and produces a wider range of responses than was thought possible even just a few years ago. Specifically, this new work shows that the fly immune response is edited and repressed. These newly discovered systems provide us with an opportunity to study both immune signaling and the pathology that can result from unregulated immune responses, and they promise to teach us about mechanisms that might be translated back to human health.

## Two Major Pattern-Recognition Pathways Control Fly Immunity

In the fly, two well-described signaling pathways (Toll and imd) respond to microbial elicitors and induce the transcription of antimicrobial peptide (AMP) genes [2]. Experiments that analyzed infections in mutant flies showed that Toll signaling is required for fighting some fungi, viruses, Gram-positive bacteria, and Gram-negative bacteria and that the imd pathway is involved in fighting some Gram-positive and Gram-negative bacteria. Many types of microbes and microbial components remain to be tested. Additionally, some unexpected elicitors have been identified; for example, sex peptides in ejaculate can induce the immune response in the female Drosophila genital tract in a Toll- and imd-dependent manner [3].

**Figure 1:**
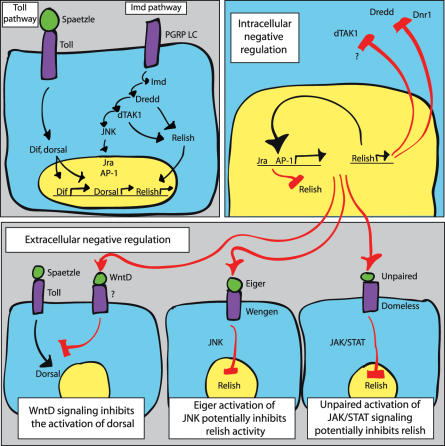
Intracellular and Extracellular Negative Regulatory Circuits Immune activation by peptidoglycan stimulates the Toll and imd signaling pathways, inducing the translocation of the transcription factors Dorsal, Dif, and Relish into the nucleus (left panel). This activates an immediate transcriptional response to infection. This response can be attenuated by cell-autonomous intracellular signals (right panel) or cell–non-autonomous extracellular signals (bottom panel). All negative regulatory circuits are highlighted in red. Note that the Toll and imd pathways are shown in a simplified form and not all of the components are listed.

Signaling through the Toll and imd pathways leads to the activation and translocation of NF- B–like proteins into the nuclei of activated cells (Figure 1). In the case of Toll signaling, the NF- B homologs Dorsal and Dif are activated. The imd pathway activates the NF- B homolog relish. Induction of imd signaling also leads to the activation of JNK signaling through the kinase dTAK1. This ultimately leads to the activation of the two transcription factors, jun-related antigen (Jra) and AP-1. Immune induction in the fly is most commonly characterized by the massive induction of antimicrobial gene transcription, although many other genes are affected both positively and negatively during an immune response. When flies are challenged with a nonpathogenic elicitor (a molecule or microbe that activates immune signaling but doesn't cause disease), this transcriptional response starts, peaks, and then resolves. Focus on this pattern recognition–driven immune response has led to a cell-autonomous view of fly immunity in which microbial elicitors induce a cell to respond in the absence of other signals.

Two important questions remained unanswered by this early work: “How and (perhaps more importantly) why does the immune system turn off this activated response?” A simple answer to “how?” might be that the immune response turns off when the elicitors are cleared. However, work in the last 3 years suggests that the story is more complicated and that several layers of negative regulation actively turn down immune signaling. The answer to “why?” appears to be that the immune response causes pathology when it is hyper-activated.

## Negative Regulation of Fly Immunity Can Occur through the Clearance of Elicitors

At a very basic level, one might view the clearance of microbes as a negative regulator of the immune response—if the microbes are gone, there will be no more inducers and the immune response is no longer activated. Physical clearance of microbes occurs rapidly in flies through the action of phagocytic cells called hemocytes. This story is complicated because, after phagocytosis (the ingestion of particulates), hemocytes release unknown signals that activate a systemic immune response [4]. Phagocytes thus remove one signal and, at the same time, produce secondary activating signals.

Clearance of subcellular microbial elicitors follows a similar pattern. The bacterial cell wall component peptidoglycan is the chief bacterial elicitor known to activate Toll and imd signaling; amidases that degrade peptidoglycan likely help clear this material from the circulation. Mutant flies lacking these enzymes induce more AMP transcription [5,6]; again, this story is complicated because enzymes that degrade peptidoglycan also release soluble potent immune elicitors from insoluble peptidoglycan and may be required for some immune responses, like phagocytosis [7].

## Negative Regulation of Fly Immunity Can Occur within and between Cells

Besides this clearing activity, both cell-autonomous and cell–non-autonomous timing mechanisms exist to turn down immune responses. Toll signaling can be repressed by a Toll-regulated secreted inhibitor. Toll activation results in the transcription of wntD, a member of the wnt family of signaling ligands [8,9], forming a simple negative regulatory circuit whose timing depends on the kinetics of signaling through both the Toll and wntD pathways. The ligand wntD signals through an unknown pathway to block nuclear translocation of the transcription factor Dorsal; as a result, wntD mutants produce higher levels of some antimicrobial peptides. These mutants have a counterintuitive immune phenotype; they are more sensitive to infections by the pathogenic bacterium Listeria monocytogenes. This is surprising because an increased immune response might be expected to lead to increased survival during an infection. In most cases, we don't understand why flies die when they are infected. It is possible that the increased production of AMPs uses energy that might otherwise be important for survival or that the immune response itself could be pathogenic.

The imd pathway is also controlled by a simple negative-feedback loop. Activation of the imd pathway induces transcription of the conserved *gene defense repressor 1 (dnr1)* [10]. Dnr1 is a negative regulator of the caspase Dredd, a component of the imd signaling pathway. Presumably, as Dnr1 concentrations rise, the imd pathway is shut down.

Activation of the imd pathway leads to signaling through both the imd and JNK signaling pathways, and both pathways negatively regulate each other. Imd regulates JNK signaling by inducing genes downstream of relish that cause the degradation of the kinase dTAK1 [11]. Activation of the JNK pathway leads to down-regulation of relish activity, which is mediated by a protein complex including the JNK target AP-1 as well as histone deacetylase [12]. The final result is that both JNK and imd signaling flux should be reduced by these cell-autonomous negative regulatory circuits.

This inhibition of imd signaling through the JNK pathway suggests that there might be communication between cells leading to immune inhibition. The JNK pathway can be activated by Eiger, the sole tumor necrosis factor (TNF) homolog in the fly [13–15]. In vertebrates, many TNFs are soluble, secreted factors that are important regulators of immunity. Eiger itself is a secreted immune-induced molecule [16,17]. One can easily imagine a circuit in which eiger released by one cell limits the induction of imd signaling in another. An Eiger-immune phenotype suggests that this occurs: eiger mutants produce more imd regulated AMPs than do wild-type flies, as would be expected if a negative regulator were removed [18].

New research by Kim and colleagues published in this issue of *PLoS Biology* [19] builds on their past work showing that JNK signaling inhibits the imd pathway. The authors suggest that another extracellular signaling pathway, the JAK/STAT pathway, also inhibits imd signaling. Using cultured cells, they showed that the transcription factors Jra and Stat93E, which act downstream of the JAK/STAT and JNK pathways, can bump Relish off a promoter, thereby terminating AMP gene transcription and repressing the output of the imd pathway. This effect depends on the concentration of transcription factors, which increases in the nucleus during immune activation. Both Jra and Stat93E are regulated by signaling pathways that are induced by extracellular signals. The authors demonstrated that STAT activation required the presence of the gene *domeless*, which expresses the receptor in the fly JAK/STAT pathway. The requirement for JAK/STAT signaling in addition to Stat93E concentration suggests that this negative regulatory switch is sensitive to extracellular signals. The Domeless-JAK/STAT signaling pathway is the fly homolog of vertebrate cytokine signaling pathways, which are involved in both the positive and negative regulation of human immune responses [20].

By moving from a mechanistic description of this signaling circuit in cultured cells to a phenotypic description in whole flies, the authors demonstrated that reduced Jra or Stat93E levels resulted in increased AMP production in whole flies during an immune response. As was seen in wntD mutant flies, these mutants transcribed more AMP transcripts but were more sensitive to infections; thus there are now several examples demonstrating that appropriate down-regulation of the immune response is important for the survival of flies during an infection.

We can now start to put this information together and imagine the progress of an infection in the fly. Cells at the initial site of infection likely respond by producing AMPs, nitric oxide, releasing bacterial elicitors of innate immunity, and synthesizing other signaling molecules. Multiple factors (the amount of microbial material, how it spreads, and the pathology it produces) will produce both positive and negative immune regulators that will color the systemic response of the rest of the fly. Thus a fly does not receive a single signal (“fungus here!”) nor does it robotically respond (“turn on AMP expression”); rather, a mixture of signals, in combination, allow more nuanced alarms (“fungus present, but we've got this under control, don't waste energy fighting that infection” or “put everything we've got into defense now or we are going to die!”) permits more complex, spatially, and temporally regulated responses.

## Abbreviations

AMP: antimicrobial peptide
